# Land Fragmentation, Technology Adoption and Chemical Fertilizer Application: Evidence from China

**DOI:** 10.3390/ijerph19138147

**Published:** 2022-07-02

**Authors:** Liang Chi, Shuqing Han, Meili Huan, Yajuan Li, Jifang Liu

**Affiliations:** 1 Agricultural Information Institute, Chinese Academy of Agricultural Sciences, Beijing 100081, China; chiliang@caas.cn (L.C.); hanshuqing@caas.cn (S.H.); liujifang@caas.cn (J.L.); 2 China Institute for Rural Studies, Tsinghua University, Beijing 100084, China; 3 National Academy of Agriculture Green Development, Key Laboratory of Plant-Soil Interactions, Ministry of Education, College of Resources and Environmental Sciences, China Agricultural University, Beijing 100193, China; liyajuancau@cau.edu.cn

**Keywords:** land fragmentation, agricultural mechanization, ICT’s, soil testing fertilization, sustainable agricultural practices

## Abstract

Although it has been widely recognized that land fragmentation has increased chemical fertilizer application, little is known about the role of technology adoption in mitigating these adverse effects. To empirically examine the relationship between land fragmentation, technology adoption and chemical fertilizer application, we developed a mediation model. We applied our analysis to a survey data set encompassing 1388 farm-level samples collected in 14 Chinese provinces in 2019. Our study demonstrated that land fragmentation can not only directly increase chemical fertilizer application but also indirectly increase it by hindering the adoption of agricultural mechanization technologies (AMT’s) and soil testing fertilization technologies (STFT’s). Both are recognized as potent drivers of fertilizer use reductions. Moreover, the adoption of information and communications technologies (ICT’s) can help mitigate the negative effects of land fragmentation on technology adoption, thus reducing chemical fertilizer application intensity (CFAI). However, the direct effects of land fragmentation on CAFI was unaffected by ICT’s. Our findings suggest that ICT’s have revolutionized farmer recognition, promotion and adoption of agricultural technologies by increasing awareness and diffusion of agricultural technology information.

## 1. Introduction

Chemical fertilizers are widely adopted in agricultural production and play a significant role in increasing yields of agricultural products and ensuring food security [1]. However, the excessive use of chemical fertilizer has resulted in various problems such as food insecurity, soil degradation and greenhouse gas emissions in developing countries, especially in China [2]. More importantly, the overuse of chemical fertilizer in agricultural production has become a public concern, for social well-being and ecological balance are seriously threatened by massive chemical fertilizer use [3,4].

China’s agricultural production features small-scale farming and severe land fragmentation. About 210 million rural households in China operate on cultivated land less than 10 mu (0.667 hectares) and the average farm size is only 7.46 mu (0.497 hectares) [5]. Compared with other Asian countries, the farm size in China is about one-third of that in South Korea and one-quarter of that in Japan [6]. Farm households have been the driving force of agricultural production since the implementation of the Household Contract Responsibility System in 1979.

Meanwhile, China is also the country with the largest amount of chemical fertilizer application in the world in terms of overall tonnage [7]. The agricultural growth in China depends heavily on the use of chemical fertilizer. The total agricultural output increased by 42.23% from 1978 to 1984, among which 45.79 percent of this output growth came from increases in inputs, including cultivated land, labor, fertilizer and capital, and fertilizer alone contributed 32.2% of the growth [8]. Some studies suggest that the extensive use of chemical fertilizers and other inputs is the fundamental reason for the rapid growth of Chinese agriculture [9]. Consequently, the development of sustainable agriculture in China is faced with severe challenges.

The unfavorable natural resource conditions have made it essential for China to develop intensive agriculture. However, the excessive and inefficient use of agricultural inputs were quite commonly seen at the early stages of agricultural production so as to ensure food security [10,11,12,13,14]. As a result, the extensive use of agricultural inputs has greatly damaged the environment [15,16,17,18,19].

**Figure 1:**
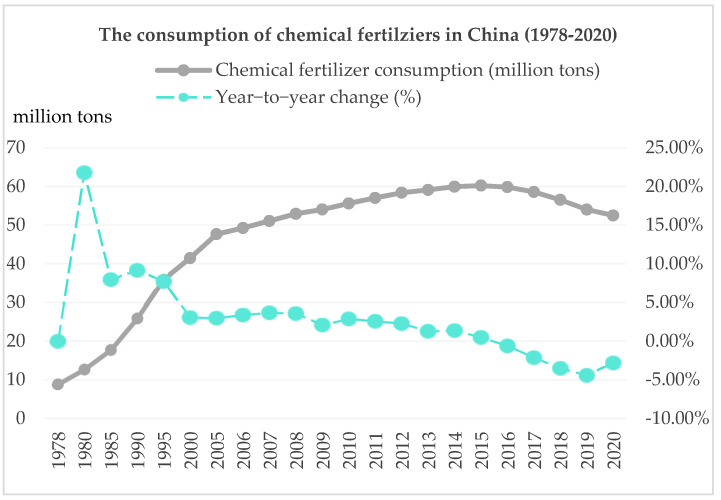
The consumption of chemical fertilizers in China from 1978 to 2020. Source of data: National Bureau of Statistics of China, 2021.

In order to reduce the use of chemical fertilizers, the Chinese government has implemented a series of policies, such as the removal of subsidies for chemical fertilizers and the promotion of soil testing technologies [20,21]. Although early studies assume that these policies may not significantly decrease chemical fertilizer application [7], we believe that these policies have helped reduce the amount of chemical fertilizer application. According to the data from National Bureau of Statistics of China [22], the consumption of fertilizers in China has seen a steady increase since 1978, reached its peak at 60.33 million tons in 2015, and started to decrease thereafter, as is shown in Figure 1.

Although there has been a slight decrease in recent years due to the policies implemented, the consumption of chemical fertilizers remains large. In 2020, there were still 52.5 million tons of chemical fertilizers consumed. Moreover, the household-level survey data from the Research Center of Rural Economy (RCRE) of the Ministry of Agriculture and Rural Affairs of China shows a similar trend. The survey dataset with 17,000 farm-level observations in 31 provinces of China shows that the amount of chemical fertilizers applied by Chinese farmers basically remained at 464.18 kg/ha from 1995 to 2015 [6]. A question arises: what are the root causes of small farmers applying such an enormous amount of chemical fertilizer?

To answer this question, a growing body of literature has explored the drivers of chemical fertilizer application. The results, however, are unclear and even conflicting. While several studies suggested that farm household and farmer characteristics, including, farm size, cropping structure and resource endowment, have significant effects on the amount of chemical fertilizer application [23,24,25,26,27], others show that the effects of individual characteristics on fertilizer application tend to be weakened over time since smallholder farmers are likely to imitate each other and apply the same amount of fertilizers [28,29,30]. Therefore, land resource conditions are still regarded as one of the key drivers for chemical fertilizer application. In particular, China is faced with unfavorable land resource endowment, such as extremely small farm size and serious land fragmentation and whether it restricts the reduction of fertilizer application has raised a lot of concern.

Most of the existing studies investigating the impact of natural resource endowment on fertilizer use are mainly focused on farm size [31,32], and little is known about whether the characteristics of farmland affect chemical fertilizer use, and the influence mechanism remains unclear. Some studies in the literature have argued that land consolidation through land use rights circulation contributes to the reduction of chemical fertilizer use [33]. However, land use rights trading may increase the degree of land fragmentation, which increases the difficulty in agricultural production and farm management. It is unreasonable to discuss farm size only and ignore the role of land fragmentation. Furthermore, the results of the studies on the relationship between farm size and fertilizer use are unclear and even conflicting. While some studies show that increasing farm size can reduce chemical fertilizer application without decreasing or even increasing crop yield [7,31], others find that smaller farm size can lead to higher fertilizer use efficiency [32]. Moreover, precious few studies have explored the negative impact of land fragmentation on farmers’ fertilizer use efficiency and discussed the heterogeneous effects of different contributing factors, such as farm size, crop structure and land quality [34]. However, the influence mechanism has not yet been fully understood.

More importantly, the existing literature has shown that land fragmentation may hinder the adoption of modern agricultural machinery [35], increase production costs [36] and cause the loss of technical efficiency [37] and land use efficiency [38]. Hence, land fragmentation may also have a direct impact on farmers’ behavior regarding chemical fertilizer application. On one hand, instead of using machinery, smallholder farmers are likely to increase other inputs such as applying more chemical fertilizers and using more labor since land fragmentation increases the difficulty of mechanical operation, resulting in higher mechanical costs [39,40]. In particular, the low ratio of fixed inputs to total inputs is the key factor leading to over-fertilization on smallholder farms because smallholders lack fixed inputs and then compensate by over-applying fertilizer to attempt to achieve their yield goals [41]. On the other hand, land fragmentation also makes it possible for farmers to flexibly distribute labor and other inputs and thus improve efficiency [42,43].

Based on the above observations and previous studies, we hypothesize that land fragmentation has a significantly positive effect on chemical fertilizer application, and the adoption of agricultural technologies plays an important role in it. In other words, land fragmentation exerts a significant influence on farmers’ chemical fertilizer application via its influence on the adoption of agricultural mechanization technologies (AMTs) and soil testing fertilization technologies (STFTs), and the information and communications technologies (ICTs) can help mitigate these negative effects. To fill in the literature gap, in this study, we provide a robust estimation of the effects of land fragmentation on farmers’ chemical fertilizer application as well as the role of the adoption of three technologies in China’s maize production.

The objectives of this study are two-fold. The first is to explore how land fragmentation and the adoption of two agricultural technologies, i.e., AMT and STFT, affect chemical fertilizer application intensity (CFAI) in maize production through a mediation model. The second is to investigate how ICT adoption mitigates the negative effects of land fragmentation on the adoption of two agricultural technologies and the reduction of chemical fertilizer application. Our analysis reveals the mechanism by which land fragmentation affects farmer’s chemical fertilizer application via agricultural technology adoption. Specifically, land fragmentation changes the adoption of AMTs and STFTs, resulting in increasing farmers’ chemical fertilizer application. Moreover, the adoption of ICTs can mitigate the process where land fragmentation negatively affects AMT and STFT adoption. To our knowledge, this study is among the first to investigate the effects of land fragmentation on chemical fertilizer application through the adoption of three technologies in rural China and therefore help shed light on the issue. Our study also has important implications for developing countries with agricultural characteristics similar to China.

The remainder of this paper is organized as follows. In Section 2 we provide the data and estimation strategy, followed by the estimation results in Section 3. Section 4 presents the discussion, and Section 5 concludes.

## 2. Methodology and Data

### 2.1. Empirical Model

#### 2.1.1. Chemical Fertilizer Application Intensity

Chemical fertilizer application intensity (*CFAI*) is measured as the consumption of chemical fertilizers per hectare sown area. It is a general index to reflect chemical fertilizer use and corresponding ecological risks [44]. The *CFAI* can be calculated as:

(1) $$CFAI=\frac{CCF}{SAC}$$

where *CCF* denotes the consumption of chemical fertilizers, which refers to the total amount of chemical fertilizers applied in maize production, including nitrogenous fertilizers, phosphate fertilizers, potash fertilizers and complex fertilizers. *SAC* denotes the total sown area of crops, which includes the land owned by the farmers themselves and that transferred from others.

#### 2.1.2. Simpson’s Index of Diversity

Before the assessment, we needed an indicator containing all important factors to measure the degree of land fragmentation. Three indicators are widely used in the existing literature to measure the degree of land fragmentation, i.e., number of plots, average plot size, average plot distance [31]. The Simpson Index of Diversity (*SI*), a general indicator to represent land fragmentation [45,46], is defined as:

(2) $$SI=1-\frac{\sum_{i=1}^{n}a_{i}^{2}}{{\left(\sum_{i=1}^{n}a_{i}\right)}^{2}}$$

where 0≤SI≤1, when SI=0, which means that the household has only one piece of land, with a higher value of SI indicating a higher degree of land fragmentation. n is the number of plots that the household has. $a_{i}$ is size of plot i.

#### 2.1.3. Plot Distance Index

However, the Simpson index does not capture the distance of each plot [46]. Hence, we constructed a plot distance index (*PDI*) which captures the spatial distribution of plots of the farm household. The *PDI* is defined as:

(3) $$PDI=\frac{d_{1}}{d_{max}}\times \frac{d_{2}}{d_{max}}\times \frac{d_{3}}{d_{max}}\times \cdots \times \frac{d_{n}}{d_{max}}$$

where $d_{i}$ denotes the distance between the farmer’s house and the plot *i.* $d_{max}$ is the distance of the farthest plot to farmer’s house, with a larger value of *PDI* indicating a higher degree of land fragmentation.

#### 2.1.4. Mediation Model

In order to examine the mechanism of how land fragmentation affects *CFAI*, we employed a mediation model to explore if agricultural technology adoption mediates the effect of land fragmentation on *CFAI*. Here, we categorize agricultural technology adoption into two types, *AMT* and *STFT*. The mediating effect mainly tests the role of agricultural technology adoption in facilitating the process through which land fragmentation affects *CFAI*. The three-model system is widely used to examine the mediating effects of mediators [47], and we set up the three-model system as follows:

(4) $$CFAI_{i}=\gamma_{0}+\gamma_{1}SI_{i}+\gamma_{2}X_{ki}+\varepsilon_{1i}$$

(5) $$M_{i}=a_{0}+a_{1}SI_{i}+a_{2}X_{ki}+\varepsilon_{2i}$$

(6) $$CFAI_{i}=\rho_{0}+\rho_{1}SI_{i}+\rho_{2}M_{li}+\rho_{3}X_{ki}+\varepsilon_{3i}$$

Here $SI_{i}$ indicates the Simpson index of farm i; $M_{i}$ is the mediator, namely, *AMT* adoption and *STFT* adoption of farm i; $X_{ki}$ is a vector of other variables affecting agricultural technology adoption and *CFAI*, including factors such as farm household and farmer characteristics, farmland characteristics, region characteristics, policies, etc., following the existing studies [11,37,48]. $\varepsilon_{i}$ is a random error term.

Specifically, we first test the direct effects of land fragmentation on *CFAI* without considering technology adoption in Equation (4). Then we explore the effects of land fragmentation on agricultural technology adoption in Equation (5). The last step is to investigate the effects of land fragmentation and technology adoption on *CFAI* in Equation (6). If we find $a_{1}$ equal to 0, $\rho_{2}$ equal to 0, or $\rho_{1}$ equal to $\gamma_{1}$, then we cannot reject the null hypothesis that there is not a mediating effect.

To better understand the role of *ICT* adoption in the relationship among land fragmentation, agricultural technology adoption and *CFAI*, we introduce a dummy variable (whether the farm household uses smart phone or personal computer (*PC*) to access information about agricultural production and selling via internet) to investigate whether *ICT* adoption mitigates the negative impacts of land fragmentation on agricultural technology adoption and *CFAI*. Both this dummy variable and its interaction with land fragmentation are incorporated into the regression so that:

(7) $$CFAI_{i}=\gamma_{0}+\gamma_{1}SI_{i}+\gamma_{2}X_{ki}+\gamma_{3}ICT_{i}+\gamma_{4}SI_{i}\times ICT_{i}+\varepsilon_{1i}$$

(8) $$M_{i}=a_{1}+a_{2}SI_{i}+a_{3}X_{ki}+a_{4}ICT_{i}+a_{5}SI_{i}\times ICT_{i}+\varepsilon_{2i}$$

(9) $$CFAI_{i}=\rho_{0}+\rho_{1}SI_{i}+\rho_{2}M_{li}+\rho_{3}X_{ki}+\rho_{4}ICT_{i}+\rho_{5}SI_{i}\times ICT_{i}+\varepsilon_{3i}$$

where the dummy variable $ICT_{i}$ takes a value of “1” if the farm uses smart phone or PC, and “0” otherwise. The internet use can help reduce chemical fertilizer use [49]. Hence, we hypothesize that *ICT* adoption has significant and negative coefficients, $\gamma_{3}$, and $\rho_{4}$, in Equations (7) and (9), respectively. Additionally, *ICT* adoption enables farmers to access more information about newly developed agricultural technologies and thus mitigate the negative effects of land fragmentation on both agricultural technology adoption and reduction of chemical fertilizer use. We therefore expect that $\gamma_{4}<0$ in Equation (7); $a_{4}$ > 0, $a_{5}>0$ in Equation (8); and $\rho_{5}<$0 in Equation (9).

### 2.2. Data

This study utilizes a dataset which was obtained by a face-to-face questionnaire survey administered by the National Agricultural and Rural Development Research Institute (NARI) of China Agricultural University (CAU) in 2019. The survey mainly focuses on grain production. Multistage sampling was employed for data collection. First, 14 provinces were chosen. Second, the towns were selected in each province based on the cultivated area of grains; that is, the sample towns should produce grains. Then 1–2 villages were randomly selected from each town. Next, 15–20 farm households were chosen from each village. As there might be farm households that are reluctant to participate in the survey, such a household would be replaced by another household.

From November to December of 2018, the NARI recruited students from CAU and trained them to guarantee that these students can collect appropriate data during the survey. The survey was conducted from January to February in 2019 when the university was on winter vacation. In the end, 2866 farm-level questionnaires from grain growers were obtained, covering a total of 14 provinces. The heads of the farm households were asked to answer the questionnaire based on their farm management in 2018. The survey data provide information on the inputs and outputs of crop production, land, income, expenditure and farm household characteristics.

**Figure 2:**
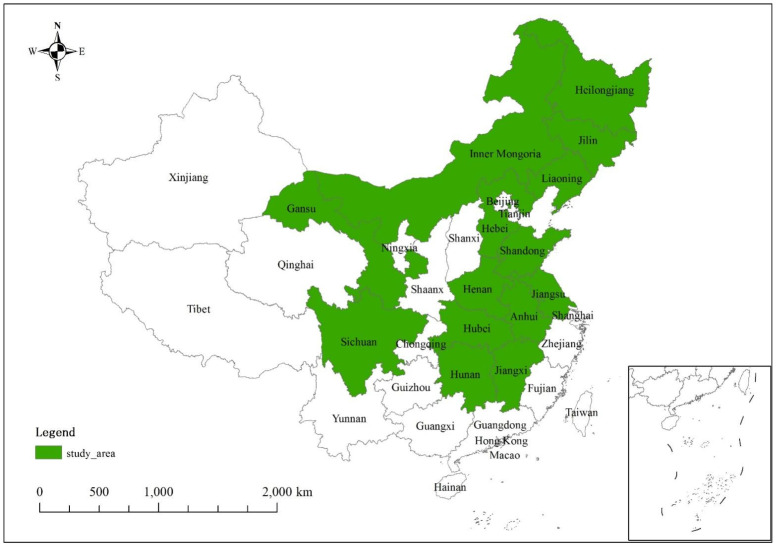
Profile of study areas.

Since our study is focused on smallholder farmers, we excluded the observations from farm sizes more than 2 hectares, according to classification by the World Bank. Additionally, inconsistent and incomplete questionnaires were dropped. The final dataset consists of 1388 farm households engaging in maize production, covering 144 villages from 119 counties across 14 provinces, namely Inner Mongolia, Jilin, Sichuan, Anhui, Shandong, Jiangsu, Jiangxi, Hebei, Henan, Hubei, Hunan, Gansu, Liaoning and Heilongjiang provinces, as shown in Figure 2.

The distribution of the 1388 observations from the 14 provinces is shown in Table 1. As one might want to know why there are only a few observations in Heilongjiang and Liaoning provinces, two major grain-producing provinces in China, the main reason is that most of the farms in these regions are larger than 2 hectares and were excluded from our analysis.

### 2.3. Variables and Descriptive Statistics

The dependent variable is CFAI measured as kilograms per hectare. We calculated the CFAI using the consumption of chemical fertilizers per hectare sown area. Our core independent variable is land fragmentation, measured as Simpson’s index. To provide robustness check, we used plot distance index (PDI), in the estimation.

Covariates in each equation are listed and explained in Table 2. For the two types of agricultural technology adoption, AMT is measured by a dummy variable, whether the household used agricultural machinery, and STFT is also measured by a dummy variable, whether the household adopted soil testing fertilization technologies. As one of our aims is to evaluate the moderating effects of ICT adoption on the relationship among land fragmentation, agricultural technology adoption and CFAI, we used a dummy variable, whether the household adopted ICTs, to measure ICT in our analysis.

In the mediation model, we control for farm household and farmer characteristics, such as age, gender, education, social capital, technical guidance, fixed assets investment and region characteristics, following the existing literature [6,32,50]. Characteristics of farmland, such as the terrain and structure of cropland (evaluated by flat land ratio, sloped land ratio, hilly land ratio, paddy land ratio, and dry land ratio), are also included as they are considered as crucial factors affecting household decisions regarding farming techniques [48,51,52,53]. Self-rated quality of cropland may also affect farmers’ production decisions due to the endowment effects. Therefore, a variable for self-rated quality of cropland is included in the model. Moreover, since tenure security contributes to the reduction of chemical fertilizer use [54,55], a variable for land use rights certification is included in the CFAI equation. In addition, the rural–urban migration experience is conducive to reducing fertilizer use [56], so we control for the labor migration variable. Agricultural subsidies also reduced fertilizer use by promoting the adoption of agricultural techniques, a variable that indicates whether the farm received maize producer subsidy is included in the mediation model [57].

A statistical description of variables is presented in Table 2. The average CFAI is 335.89 kg/ha, which is very large compared with some developed countries such as the United States and Japan. Moreover, the average SI is 0.68 and the average PDI is 0.18, which means that land fragmentation is severe in China. In addition, more than half of the farms have adopted AMTs and ICTs, accounting for 71% and 58.8% of the total farms, respectively. However, only 23% of the farms have adopted STFTs, implying that the advantages of sustainable agricultural technologies have not yet been fully recognized.

## 3. Estimation Results

### 3.1. Baseline Regression

**Figure 3:**
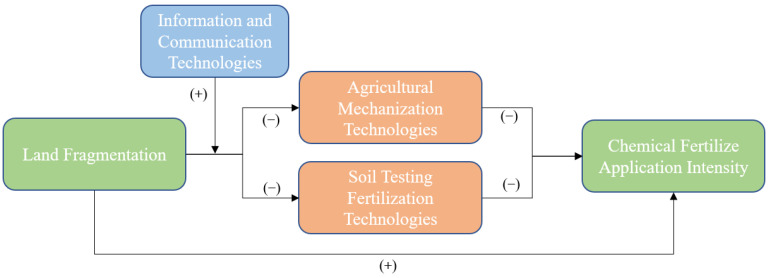
Influence mechanism of technology adoption on land fragmentation and CFAI.

Based on our observation and previous studies, we establish a conceptual framework, considering the role of AMT, STFT and ICT adoption on the effects of land fragmentation on CFAI. A possible mechanism is shown in Figure 3, and we examine it using the survey data.

Table 3 reports the mediating effect of STFT and AMT adoption on the relationship between land fragmentation and CFAI. It shows that the coefficient of SI on CFAI is significant and positive in columns (1), (3) and (5), implying that land fragmentation has a significantly positive effect on CFAI. Additionally, the coefficient of land fragmentation on STFT and AMT adoption is significant and negative, in columns (2) and (4), respectively, which means that land fragmentation has a negative impact on the adoption of these two agricultural technologies. Moreover, the coefficient of STFT and AMT adoption on CFAI is significantly negative in columns (3) and (5), respectively, meaning that the adoption of agricultural technologies has significantly decreased the CFAI. The results suggest the existence of the mediating effect of adopting two agricultural technologies, and the total effect mediated by the adoption of STFTs and AMTs are 11% and 29% respectively. As expected, land fragmentation can not only directly increase the CFAI, but it also indirectly increases the CFAI by decreasing the probability of farmers adopting agricultural technologies.

To examine the impact of ICT adoption on the relationship between land fragmentation and agricultural technology adoption, we apply OLS regression to Equations (5) and (7). The results are shown in Table 4.

As previously analyzed, without considering the role of ICT adoption, land fragmentation has a significantly negative effect on STFT adoption, as shown in column (1). After ICT and the interaction term of ICT adoption and land fragmentation were introduced into the regression, we can see from column (2) that both ICT and the interaction term have significant and positive coefficients, implying that ICT adoption can significantly increase the probability of STFT adoption and mitigate the negative effects of land fragmentation on STFT adoption.

To sum up, the adoption of ICTs significantly affects the effect of land fragmentation on STFT adoption, which can be explained by the typical characteristics of farmers using the internet in rural areas. Based on our field research experience, for the vast majority of farmers who obtain agricultural service information through the internet, they use instant messaging software called WeChat (Shenzhen, China). WeChat has revolutionized farmers’ technology adoption behaviors. On one hand, it pushes agricultural service information in real time. A large number of surveyed farmers subscribe to information services to receive the latest agricultural extension information and technical guidance. Internet use has significantly improved the availability of agricultural information. On the other hand, WeChat has significantly improved the intensity of farmers’ social networks. For farmers, the impact of internet use on social network intensity is mutual. Farmers with strong social networks are more inclined to use the internet, and internet use further increases the social network intensity of farmers.

This interaction has a specific impact on the adoption of STFTs by farmers. They can share the obtained agricultural technology information through instant messaging tools such as WeChat and further exchange information, which significantly enhances the dissemination of technology adoption experience. Farmers who have adopted the STFTs can share the relevant experience and effectiveness of technology adoption with other farmers who use WeChat, which significantly affects other farmers’ decisions regarding agricultural technology adoption. On the contrary, farmers who do not use WeChat have lower frequency and efficiency in agricultural technology information exchange. A field survey based on 1710 farmers in Hubei Province also confirmed that land fragmentation has a significant negative impact on the adoption of STFT by farmers with weak social networks [58].

Table 5 reports the effects of ICT adoption on the relationship between land fragmentation and AMT adoption. Without considering the role of ICT adoption, land fragmentation has a significantly negative effect on AMT adoption, as shown in column (1), which is consistent with the existing studies. After ICT and the interaction term of ICT adoption and land fragmentation were introduced into the regression, we can see from column (2) that both ICT and the interaction term have significant and positive coefficient, implying that ICT adoption can significantly increase the probability of AMT adoption and mitigate the negative effects of land fragmentation on AMT adoption.

### 3.2. Robustness Check

To provide a robustness check, we use the plot distance index (PDI) as an alternative variable of land fragmentation, following the existing studies [45,59]. As shown in Table 6, when PDI was used to replace SI as the independent variable, the results are completely consistent with the benchmark regression results, and land fragmentation significantly increases CFAI. In terms of the influence mechanism, land fragmentation has a significantly negative effect on the adoption of agricultural technologies and thus increases the CFAI.

### 3.3. Further Comparison

**Figure 4:**
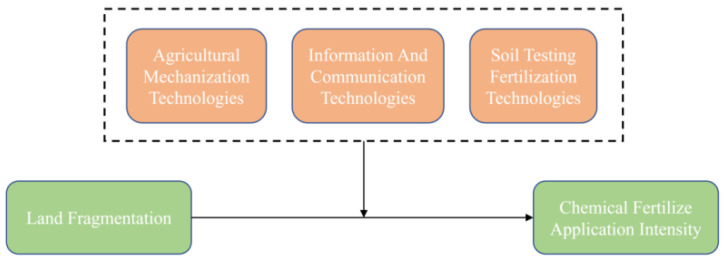
Heterogeneity in the moderating effects of the adoption of the three technologies.

To further examine the role of technology adoption on the relationship between land fragmentation and CFAI, as shown in Figure 4, we introduced the interaction term of SI and the adoption of the three technologies into the regression. The estimation results are shown in Table 7.

The results show that SI has significant and positive coefficients in column (1)–(4), implying that land fragmentation has increased CFAI. The coefficient of the interaction term of SI and AMT is significant and negative in column (3), which means that AMT adoption can mitigate the positive impact of land fragmentation on CFAI. Additionally, the results of column (4) suggest that STFT adoption can mitigate the positive impact of land fragmentation on CFAI. More importantly, the coefficient of the interaction term of SI and ICT is not statistically significant, and ICT has no significant effect on the direct effect of land fragmentation on CFAI, as shown in column (2).

Obviously, the use of agricultural machinery and the progress of agricultural technology are effective measures to reduce the intensity of chemical fertilizer use. When we examine the moderating effects of ICT on the direct effects of land fragmentation on CFAI alone, the role of information publicity and promotion brought with ICTs is limited. The unfavorable land resource endowment has restricted the adoption of advanced agricultural technologies and machinery. Therefore, even if farmers recognized the negative effects of excessive application of chemical fertilizer, they would increase the input of chemical fertilizer to ensure income and output. More importantly, the influence mechanism shown in Figure 3 confirmed that the rapid development of information technology has significantly increased the probability of farmers adopting AMTs and STFTs and thus leads to the reduction of CFAI.

## 4. Discussion

### 4.1. Role of AMT Adoption

According to the baseline regression results, the impact of agricultural mechanization exceeded our research expectations, which may be related to the stage of agricultural development in China. The issue of land fragmentation in China can be traced back to the implementation of the household contract responsibility system in the late 1980s. The arrangement of land property right system has led to the problem of land fragmentation to a certain extent [36]. However, there was no better choice for China back then. Moreover, land fragmentation did not have a negative impact on China’s agricultural production and even dispersed agricultural risks and improved the utilization efficiency of the labor force [60,61,62]. However, with the rise of labor costs and popularization of agricultural machinery, land fragmentation has increased the commuting time between plots, which limits the application of large-scale agricultural machinery. It is rather difficult for small farms to adapt to the development of modern agriculture in China. Furthermore, compared with the adoption of STFTs, whether farmers use formula fertilizer and the proportion of formula fertilizer also have significant effects on CFAI. However, due to data availability, we cannot further verify the effect of actual use of formula fertilizer on CFAI in this study.

In fact, the application of chemical fertilizer by small farmers usually remains stable in the long run [6] since it is greatly affected by previous experience. It seems like another reasonable explanation for the effects of the adoption of agricultural technology. Before the promotion and popularization of chemical fertilizer, based on the fact that the soil nutrients of cultivated land were low, China carried out chemical fertilizer efficiency tests at the national level for several years and formulated the standards for the application amounts of chemical fertilizer to grain crops according to the test results. However, farmers’ fertilization behavior is inertial and has a cumulative effect on fertilizer application. When there is no external change, such as the application of advanced technology, farmers are likely to overuse chemical fertilizer according to their experience and fertilization habits.

From the perspective of costs and benefits, from 1978 to 2014, the average annual growth rate of China’s agricultural means of production prices was 5.4%, while it was 6.4% for agricultural producer prices. It is thus profitable for farmers to increase inputs [63]. However, China’s unique urban and rural dual system and land system has diminished the advantages of China in competition with other countries with similar resource endowment conditions. The most predominant impact is the rise of agricultural labor costs and land rents. Moreover, with the continuous rise of the price of agricultural production inputs such as chemical fertilizer, the growth of marginal output and marginal income brought by increasing inputs has decreased significantly. Therefore, under the given cost constraints, it is feasible to reduce the input of other factors by increasing the use of agricultural machinery, which is reflected not only in the substitution of labor but also in the reduction of the input of means of production such as chemical fertilizer.

Based on the above analysis, taking into account the land management rights and other issues, land circulation and scale management aiming to improve the degree of mechanized operation are effective ways to reduce the intensity of chemical fertilizer application.

### 4.2. Role of STFT Adoption

The Chinese government began to implement policies to promote STFT adoption in 2005. After the implementation of the policy, the utilization rate of nitrogen fertilizer, phosphorus fertilizer and fertilizer addition in rice, wheat and corn was 33%, 24% and 42%, respectively, which increased by 5%, 12% and 10%, respectively. The effect of STFT adoption on improving the utilization rate of chemical fertilizer is obvious. However, our study shows that compared with AMT adoption, STFT adoption has a weaker influence on the effect of land fragmentation on CFAI. On one hand, the price of formula fertilizer is higher than that of chemical fertilizer. Although many farmers adopted STFTs because of policy incentives and financial subsidies, they did not use formula fertilizer. On the other hand, the effect of land fragmentation on AMT adoption is larger than that on STFT adoption.

Therefore, the popularization of STFTs can effectively promote the reduction of chemical fertilizer. Firstly, improve the market competitiveness of chemical fertilizers and pesticides use to produce agricultural products in accordance with scientific and reasonable methods. Secondly, increase the availability of professional production services for farmers, strengthen the promotion of soil testing fertilization and other technologies and increase the corresponding financial subsidies. Thirdly, reduce or even gradually abolish the preferential tax policies for chemical fertilizer production, strengthen the market supervision of excessive use of chemical fertilizer production products and improve the regulations on illegal production.

### 4.3. Role of ICT Adoption

In this study, we focus on internet use since smart phones and computers are quite powerful and play an increasingly important role in agricultural production. Based on our observation during field surveys, farmers can easily connect with the researchers from local agricultural research institutions by WeChat. For example, the National Green Manure Industry Technology System in China regularly records videos on the application of chemical fertilizer and green manure and provides corresponding information and technical services for farmers in rural areas through influential WeChat video subscription services.

Moreover, in recent years, Tik Tok is getting more and more popular and gaining wider influence. Considering that the education level of most of the farmers in rural China is relatively low, video is an efficient way to for them to access information. Tik Tok is quite significant because it has a large number of users in China. When we use agricultural planting technology as a keyword to search in Tik Tok, we can immediately get massive intuitive video information. It is noteworthy that these video providers do not rely on government support, and they are independent media practitioners. To our knowledge, there are millions of similar video providers who have created Tik Tok accounts China. At the same time, farmers can also communicate with these technical information providers through the comment function.

Our analysis shows that ICTs play the role of catalyst; that is, ICT adoption can slow down the positive effects of land fragmentation on CFAI by mitigating the negative impact of land fragmentation on the adoption of AMT and STFT. Apparently, ICT can also directly affect the fertilizer application intensity of farmers. According to our theoretical analysis and interviews with typical farmers, farmers are unlikely to change their decisions when they are faced with the pressure of crop yield and agricultural income, even if they fully understand the negative impact of excessive application of chemical fertilizer through ICT. Our results show the significant difference between ICT and typical agricultural technologies such as AMT and STFT. Most importantly, our study provides theoretical support for the Chinese government to formulate industrial policies to reduce the use of chemical fertilizer.

Based on the above analysis, we understand the mechanism of how ICTs affect the impact of land fragmentation on CFAI. It is noteworthy that it is not enough to rely merely on ICTs for policy encouragement and publicity without satisfying the demands of farmers through the application of agricultural technologies. The policies should be focused on supporting the development of agricultural technologies, give full consideration to the advantages of ICTs, and propagandize the important role of agricultural technology adoption in increasing productivity and efficiency so as to reduce the intensity of chemical fertilizer application.

## 5. Conclusions

In this study, we examined the relationship between land fragmentation and CFAI, and further explored the mediating effect of AMT and STFT adoption in China’s maize production. We developed a mediation model to explore the influence mechanism of land fragmentation on CFAI through AMT and STFT adoption. Considering the important role of ICTs played in agricultural production, we explored the impact of the ICT adoption on the relationship between land fragmentation, agricultural technology adoption and CFAI and conducted an empirical analysis on a farm level survey data with 1388 observations.

Our results clearly indicate that land fragmentation has a positive impact on CFAI, and the adoption of both AMTs and STFTs has a significant negative effect on CFAI; land fragmentation reduced the probability of farmers adopting these technologies. Moreover, the adoption of ICTs can significantly reduce the negative effect of land fragmentation on the adoption of AMTs and STFTs, but it did not directly affect the process of land fragmentation decreasing the CFAI.

This study contributes to a better understanding of the relationship between land fragmentation and chemical fertilizer use in China’s maize production. Moreover, the mediating effects of the adoption of AMTs and STFTs on the relationship between land fragmentation and chemical fertilizer use can provide insights on the influencing mechanism of land fragmentation, which is especially crucial to provinces suffering high chemical fertilizer application intensity. More importantly the adoption of ICTs can mitigate the negative impact of land fragmentation on technology adoption, which helps shed light on the issue of the low adoption rate of agricultural technologies in rural China. Therefore, policies should be carried out to continue to strengthen the extension, promotion and adoption of agricultural technologies such as AMTs and STFTs. In addition, it is of significance to give full consideration to the role of information technologies and to promote technology adoption in rural China.

The generalization of the findings of this study is subject to certain limitations. For example, the study was limited to maize production in 14 provinces in China. The results may not be able to be applied to other areas of grain production in the whole nation. China is a diverse country in terms of varying crop varieties and economic development across regions. Studies on other crops and other regions can be conducted to enrich the study in this field.

## Figures and Tables

**Table 1:** Distribution of observations by province/autonomous region.

Province	N	Percentage (%)
Inner Mongolia	82	5.91%
Jilin	69	4.97%
Sichuan	166	11.96%
Anhui	38	2.74%
Shandong	257	18.52%
Jiangsu	110	7.93%
Jiangxi	74	5.33%
Hebei	139	10.01%
Henan	200	14.41%
Hubei	111	8.00%
Hunan	58	4.18%
Gansu	15	1.08%
Liaoning	39	2.81%
Heilongjiang	30	2.16%
Total	1388	100.00%

**Table 2:** List of variables and definitions.

Variable	Definition and Descriptions	Mean	Std. Err.
Dependent Variable			
CFAI	Continuous variable, chemical fertilizer application intensity in maize production (kg/ha), measured using the CCF divided by SAC, expressed as a natural log (ln)	9.82	4.81
Variables of Interest			
SI	Continuous variable, land fragmentation, measured as Simpson’s Index of Diversity	0.68	0.22
PDI	Continuous variable, plot distance index, proxy of land fragmentation, used for robustness test	0.18	0.36
AMT	Dummy variable, agricultural mechanization technology, “1” if the farm household used agricultural machinery during production, i.e., tillage, sowing, pest control, irrigation or harvesting, “0” otherwise	0.71	0.35
STFT	Dummy variable, soil testing fertilization technology, “1” if the farm household adopted the soil testing fertilization technology before the application of chemical fertilizer, “0” otherwise	0.23	0.38
ICT	Dummy variable, information and communication technology, “1” if the farmer used smart phone or personal computer, “0” otherwise	0.59	0.86
Control Variables			
Chemical fertilizer price	Continuous variable, the average price of chemical fertilizer purchased by farmers in 2018 (CNY/kg), expressed as a natural log (ln)	1.66	0.27
Herbicide	Continuous variable, the quantity of herbicide input in maize production per hectare in 2018 (kg/ha), expressed as a natural log (ln)	0.58	0.13
Farm size	Continuous variable, measured as the operated area of maize cropland (hectare), expressed as a natural log (ln)	2.01	1.36
Labor migration	Continuous variable, measured as the percentage of farm household members employed in non-agricultural sector	0.43	0.49
Agricultural investment	Continuous variable, measured as the depreciation expense of fixed assets used in maize production in 2018 (CNY), expressed as a natural log (ln)	10.65	15.38
Crop structure	Continuous variable, measured as the share of sales revenue of grains in agricultural income	0.75	0.51
Sell mode	Dummy variable, “1” if the sell mode is instant sale, “0” if the sale mode is contract sale	0.23	0.42
Village leader	Dummy variable, “1” if the farmer is village leader, “0” otherwise	0.16	0.37
Flat land ratio	Continuous variable, the percentage of flat land in the total operated land area (%)	0.64	0.33
Sloped land ratio	Continuous variable, the percentage of sloped land in the total operated land area (%)	0.21	0.12
Hilly land ratio	Continuous variable, the percentage of hilly land in the total operated land area (%)	0.15	0.11
Paddy land ratio	Continuous variable, the percentage of paddy fields in the total operated land area (%)	0.09	0.06
Dry land ratio	Continuous variable, the percentage of dry fields in the total operated land area (%)	0.91	0.77
Self-rated land quality	Ordered variable, indicating the self-rated quality of the operated land, “1” if the land is barren, “2” if low quality, “3” if medium, “4” if medium to high, and “5” if extremely fertile	3.03	0.88
Land use rights	Dummy variable, “1” if the land use rights were registered and certificated, “0” otherwise	0.92	0.98
Age	Continuous variable, age of the household’s head, expressed as a natural log (ln)	3.94	0.23
Education	Ordered variable, education level of the household’s head (1–6), “1” illiterate, “2” elementary school, “3” middle school, “4” high school or vocational high school, “5” three-year college, and “6” college or post-graduate	2.76	0.95
Male	Dummy variable, “1” male, “0” female	0.77	0.42
Social capital	Continuous variable, measured as the frequency of the farms reach out to their friends, i.e., the number of friends or relatives the household says hi to via WeChat, phone calls or meetings during spring festival, expressed as a natural log (ln)	4.00	4.35
Technical guidance	Dummy variable, “1” if the farm household received technical guidance, “0” otherwise	0.16	0.27
Cooperative	Dummy variable, “1” if the farm household is member of cooperatives, “0” otherwise	0.25	0.41
Fixed assets investment	Continuous variable, measured as the depreciation expense of total fixed assets in 2018 (CNY), expressed as a natural log (ln)	9.27	11.32
Hired labor ratio	Continuous variable, measured as the number of work days of hired labor divided by the total number of work days devoted to maize production in 2018	0.12	0.54
Inward land transfer	Dummy variable, “1” if the farm household leased farmland from others, “0” otherwise	0.14	0.28
Outward land transfer	Dummy variable, “1” if the farm household transferred land use rights to others, “0” otherwise	0.02	0.12
Producer subsidy	Dummy variable, “1” if the farm household received a subsidy on maize production, “0” otherwise	0.15	0.36
Machinery subsidy	Dummy variable, “1” if the farm household received a subsidy on the purchase of agricultural machinery, “0” otherwise	0.05	0.22
East	Dummy variable, “1” if farm household is located in eastern region, “0” otherwise	0.34	0.47
Central	Dummy variable, “1” if farm household is located in central region, “0” otherwise	0.53	0.50
West	Dummy variable, “1” if farm household is located in western region, “0” otherwise	0.13	0.33

Notes: 1. The sum of flat land ratio, sloped land ratio and hilly land ratio equals 1. 2. Land use rights refers to the registration and certification of farmland. In particular, the rural land registration and certification program started since the No. 1 central document in 2013 was issued. It is the confirmation of land ownership, land tenure (land use rights) and other rights. The rights of each parcel must be subject to land registration procedures such as land registration application, cadastral investigation, verification of affiliation, registration and issuance of land certificates. 3. We categorize the 14 provinces into three regions according to the geographic location. Eastern region includes Hebei, Liaoning, Jiangsu, Shandong. Central region includes Inner Mongolia, Jilin, Heilongjiang, Anhui, Henan, Hubei, Hunan, Jiangxi. Western region includes Sichuan and Gansu.

**Table 3:** Mediating effects of STFT and AMT adoption on the impact of SI on CFAI.

Variable	(1)	(2)	(3)	(4)	(5)
	CFAI	STFT	CFAI	AMT	CFAI
STFT			−0.121 **		
			(0.052)		
AMT					−0.238 ***
					(0.067)
SI	0.289 ***	−0.268 *	0.254 ***	−0.352 ***	0.205 ***
	(0.051)	(0.162)	(0.056)	(0.039)	(0.047)
					
Control	Yes	Yes	Yes	Yes	Yes
					
_cons	3.565 ***	0.233 ***	3.112 ***	0.558 ***	3.223 ***
	(1.100)	(0.089)	(0.655)	(0.110)	(0.724)
Obs.	1388	1388	1388	1388	1388
R-sqr	0.238	0.117	0.236	0.121	0.241
Sobel tests		0.0019 *		0.0022 ***	
		(0.0010)		(0.0006)	
Total effect mediated		11%		29%	

Notes: Robust errors are in parenthesis, *** *p* < 0.01, ** *p* < 0.05, * *p* < 0.1. CFAI refers to chemical fertilizer application intensity, SI refers to Simpson’s Index of Diversity, AMT refers to agricultural mechanization technology adoption, STFT refers to soil testing fertilization technology adoption.

**Table 4:** Effect of ICTs on the relationship between land fragmentation and STFT adoption.

Variable	(1)	(2)
ICT		0.644 ***
		(0.163)
SI × ICT		0.141 ***
		(0.043)
SI	−0.268 ***	−0.286 ***
	(0.162)	(0.096)
Control	Yes	Yes
_cons	0.233 ***	0.226 ***
	(0.089)	(0.077)
Obs.	1388	1388
R-sqr	0.117	0.285

Notes: Robust errors are in parenthesis, *** *p* < 0.01. The dependent variable is soil testing fertilization technology (STFT) adoption. SI refers to Simpson’s Index of Diversity. ICT refers to information and communications technology adoption.

**Table 5:** Effect of ICTs on the relationship between land fragmentation and AMT adoption.

Variable	(1)	(2)
ICT		0.552 ***
		(0.201)
SI × ICT		0.123 ***
		(0.031)
SI	−0.315 ***	−0.321 ***
	(0.072)	(0.058)
Control	Yes	Yes
_cons	0.245 ***	0.211 ***
	(0.051)	(0.063)
Obs.	1388	1388
R-squared	0.298	0.208

Notes: Robust errors are in parenthesis, *** *p* < 0.01. The dependent variable is agricultural mechanization technology (AMT) adoption. SI refers to Simpson’s Index of Diversity. ICT refers to information and communications technology adoption.

**Table 6:** Effects of PDI on CFAI mediated by STFT and AMT adoption.

Variable	(1)	(2)	(3)	(4)	(5)
	CFAI	STFT	CFAI	AMT	CFAI
STFT			−0.163 ***		
			(0.031)		
AMT					−0.240 ***
					(0.076)
PDI	0.339 ***	−0.342 **	0.283 ***	−0.440 **	0.233 ***
	(0.128)	(0.144)	(0.050)	(0.191)	(0.068)
					
Control	Yes	Yes	Yes	Yes	Yes
_cons	3.441 ***	0.258 ***	3.625 ***	0.571 ***	3.145 ***
	(0.586)	(0.044)	(0.829)	(0.010)	(0.603)
Obs.	1388	1388	1388	1388	1388
R-sqr	0.366	0.185	0.268	0.167	0.308
Sobel Mediation Tests		0.0008 ***		0.0015 ***	
		(0.0002)		(0.0000)	
Total effect mediated		16%		32%	

Notes: Robust errors are in parenthesis, *** *p* < 0.01, ** *p* < 0.05. CFAI refers to chemical fertilizer application intensity, PDI refers to plot distance index, AMT refers to agricultural mechanization technology adoption, STFT refers to soil testing fertilization technology adoption.

**Table 7:** Effects of technology adoption.

Variable	(1)	(2)	(3)	(4)
SI	0.289 ***	0.278 ***	0.285 ***	0.281 ***
	(0.096)	(0.086)	(0.070)	(0.091)
SI × ICT		−0.215		
		(0.187)		
SI × AMT			−0.176 ***	
			(0.049)	
SI × STFT				−0.121 **
				(0.056)
Control	Yes	Yes	Yes	Yes
cons	3.565 ***	3.178 ***	3.456 ***	3.923 ***
	(1.100)	(0.739)	(1.197)	(0.713)
Obs.	1388	1388	1388	1388
R-sqr	0.238	0.222	0.233	0.235

Notes: Robust errors are in parenthesis, *** *p* < 0.01, ** *p* < 0.05. The dependent variable is chemical fertilizer application intensity (CFAI). SI refers to Simpson’s Index of Diversity, AMT refers to agricultural mechanization technology adoption, STFT refers to soil testing fertilization technology adoption, STFT refers to soil testing fertilization technology adoption, ICT refers to information and communications technology adoption.

## Data Availability

The associated dataset of the study is available upon request from the corresponding author.
